# An Intramyocardial Lipoma Mimicking Post-infarction Fatty Changes: Discussion of Key Distinguishing Imaging Findings and Clinical Implications

**DOI:** 10.7759/cureus.46955

**Published:** 2023-10-13

**Authors:** Charles A Wood, Rutger S Gunther, Kevin J O'Gorman, Faith Kelly, Christopher J Lisanti

**Affiliations:** 1 Radiology, New York Institute of Technology College of Osteopathic Medicine at Arkansas State, Jonesboro, USA; 2 Nuclear Medicine/Radiology, Brooke Army Medical Center, Fort Sam Houston, USA; 3 Cardiology, Brooke Army Medical Center, Fort Sam Houston, USA; 4 Cardiology, Uniformed Services University of the Health Sciences, Bethesda, USA; 5 Radiology, Brooke Army Medical Center, Fort Sam Houston, USA; 6 Radiology, Uniformed Services University of the Health Sciences, Bethesda, USA

**Keywords:** primary cardiac tumors, low-dose computed tomography, benign cardiac neoplasms, cardiac magnetic resonance imaging, cardiac lipoma

## Abstract

Cardiac lipomas are benign primary cardiac tumors that are most often asymptomatic and diagnosed incidentally. Cardiac magnetic resonance imaging (MRI) is the imaging modality of choice when aiming to characterize these tumors. A minority of cardiac lipomas are intramyocardial, which, when combined with the much more common post-infarction fatty metaplasia, makes diagnosing these lipomas very challenging. We review a case of intramyocardial lipoma in the distal interventricular septum that was initially detected on a low-dose computed tomography for lung cancer screening and the subsequent findings on cardiac MRI that made the diagnosis. Additionally, this case also helps to support the conservative management of intramyocardial lipomas that are more distal in the left ventricle and subsequently at lower risk for conduction arrhythmias.

## Introduction

Primary cardiac neoplasms are rare in the general population. The prevalence of such tumors ranges from 0.0017% to 0.02%, with approximately 75% found to be benign [1]. Most benign tumors arising from the heart are myxomas, with lipomas accounting for 8.4% [2]. Cardiac lipomas are well-circumscribed encapsulated lesions and are best characterized by cardiac MRI [3]. On histological investigation, they are composed of mature adipocytes [3,4]. These benign tumors can arise anywhere within the heart; however, they are commonly located in the right atrium and left ventricle [1]. While most lipomas are clinically silent, the location of the mass is pertinent, as it can help guide medical management and understand potential symptomatology [3,5]. In this report, we describe a patient with an asymptomatic lipoma located within the distal interventricular septum incidentally discovered on low-dose computed tomography. Because of their low prevalence, we aim to highlight the role of cardiac MRI, specifically balanced steady-state free precession (b-SSFP) sequences, late gadolinium enhancement (LGE), and wall motion in diagnosing lipomas within the myocardium while discussing the management of these tumors.

## Case presentation

**Figure 1:**
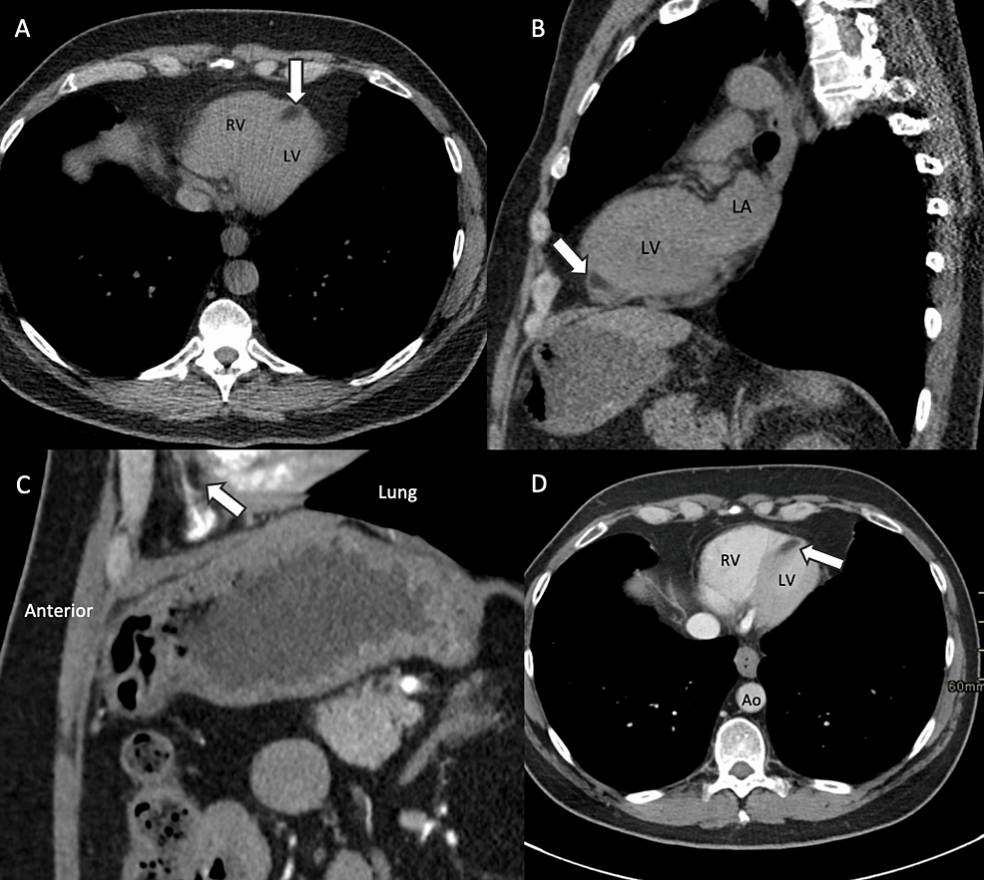
Axial (A) and sagittal (B) unenhanced computed tomography revealed a hypoattenuating fatty mass in the distal interventricular septum. Sagittal arterial phase (C) and axial venous phase (D) from a CT kidney six years prior better delineate the chambers of the heart and the position of the lipomatous mass within the septal wall. Axial arterial phase images did not reveal the lesion as clearly as sagittal images. RV: right ventricle; LA: left atrium; LV: left ventricle; Ao: aorta.

A 60-year-old male with a history of hypertension, hyperlipidemia, and a 35-pack-year smoking history with dyspnea on exertion was referred for a screening low-dose CT chest. CT scan revealed a focal fatty-attenuating region within the distal inferoseptum, which was initially thought to be lipomatous changes concerning for prior myocardial infarction given the patient’s cardiac risk factors (Figures 1A, 1B). Upon review of prior CT studies, the lipomatous lesion had been stable for six years but had not been definitively characterized (Figures 1C, 1D).

**Figure 2:**
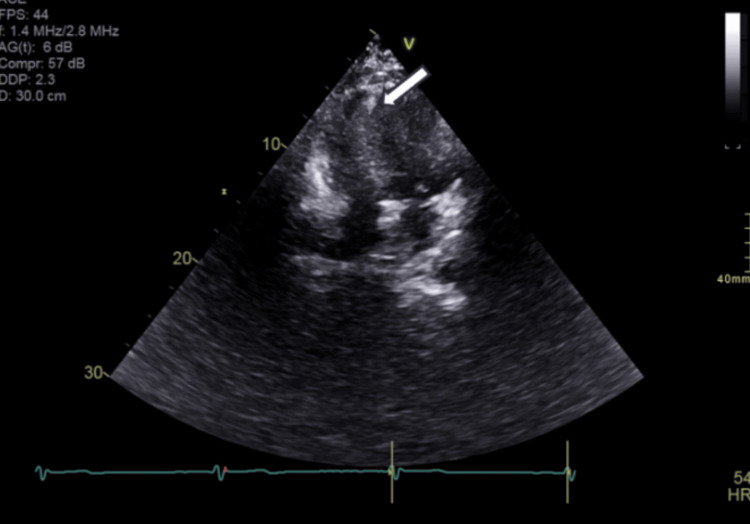
Four-chamber view of the heart on a transthoracic echocardiogram did not identify the cardiac lipoma prospectively. Retrospectively, a hyperechoic area that could represent lipoma is highlighted with an arrow.

The patient had a transthoracic echocardiogram (TTE) performed three years prior to low-dose screening CT imaging for complaints of dyspnea. TTE did not prospectively identify the lipoma; however, it appears to be seen retrospectively (Figure 2). Importantly, the study did note a normal left and right ventricular cavity size, normal left and right ventricular systolic function, and no regional wall motion abnormalities. In addition to the echocardiogram, the patient underwent a pharmacologic nuclear perfusion study that did not reveal any left ventricular abnormalities.

**Figure 3:**
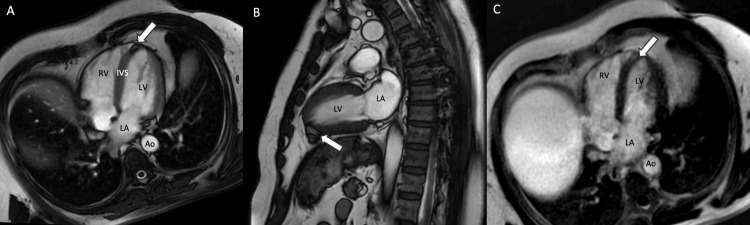
Four-chamber (A) and two-chamber (B) balanced steady-state free precession (b-SSFP) MRI images demonstrate a bright internal signal with a “black boundary” line at the interface of the lipoma within the myocardium. Four-chamber late gadolinium enhancement (LGE) image (C) shows a bright signal, which is nonspecific for infarct as fat (observe subcutaneous fat signal) or any other tissue with a short T1 time would also show a bright signal. Also, note the preservation of dark subendocardial signal adjacent to the fatty lesion. The constellation of findings from the b-SSFP images along with the CT confirms the fatty nature of the lesion rather than infarct-related LGE. RV: right ventricle; LA: left atrium; LV: left ventricle; Ao: aorta; IVS: interventricular septum.

Cardiology consultation was obtained and further evaluation with cardiac magnetic resonance imaging was performed. Of note, T1 and T1 with fat saturation sequences were not obtained in this patient because the primary clinical suspicion was fatty infiltration post-myocardial infarction. The lesion was well-circumscribed and hyperintense on b-SSFP images with a "black boundary" effect adjacent to the myocardium consistent with out-of-phase chemical shift artifact confirming the fatty composition (Figures 3A, 3B). There was no associated volume loss, myocardial thinning, or wall motion abnormality to suggest prior infarction. LGE images demonstrated a 12.3 x 10 mm intramyocardial hyperintense mass consistent with a short T1 relaxation time (Figure 3C). Overall, findings that supported a diagnosis of lipoma versus post-infarct metaplasia were (1) lack of myocardial wall thinning or wall motion abnormality, (2) preservation of dark subendocardium on the LGE sequences adjacent to the fatty lesion, and (3) absence of any other bright signal in the myocardium adjacent to the fatty lesion on the LGE sequences. Usually, fatty metaplasia post-infarct has additional surrounding LGE that extends beyond the fatty changes. Due to reassuring imaging findings including its distal location and stability demonstrated between studies six years apart, the patient was not referred to cardiothoracic surgery for resection of the lipoma.

Given the size and location of the mass, cardiology concluded a low concern for electrical (arrhythmic) complications in the future secondary to small size and distal location in the myocardium. Furthermore, future obstructive symptoms are unlikely since the lesion is intramyocardial and not subendocardial. The patient was recommended to undergo repeat TTE in three to five years to assess for changes in cardiac function related to lipoma and evaluate for any interval growth with primary care follow-up for management and optimization of his cardiovascular risk factors.

## Discussion

Cardiac lipoma is a benign tumor originating within the heart. Most cardiac lipomas arise from the subendocardial or epicardial layer of the heart, with only 25% located within the myocardium (intramyocardial) [2]. Intramyocardial masses have a predilection for causing arrhythmias as the conduction system has the potential to be affected [1,6,7]. When cardiac lipomas become large enough, particularly when they are subendocardial, their mass effect can distort entire chambers, presenting with obstructive symptoms in the patient. The most likely cardiac chamber locations for lipomas are the right atrium and left ventricle [1,3]. While cardiac lipomas can occur in any age group, there appears to be a predilection for adults ranging from 40 to 60 years old without differences in prevalence based on sex [2,3]. Symptomatic patients commonly report dyspnea, palpitations, or syncopal episodes [6]. Cardiac lipomas rarely cause embolic events [3]; however, it is reasonable to postulate that atrial arrhythmias secondary to myocardial infiltration from lipoma could promote such a phenomenon.

With the widespread use of chest CT for lung cancer screening and other indications, detecting fatty lesions in the heart is not uncommon and is usually attributed to prior myocardial infarction. However, as we demonstrate in this patient, assuming all fatty areas are a consequence of prior myocardial infarction can be misleading, and thus, correlation with prior cardiac imaging is essential to correctly diagnose the patient. If there are no substantiating studies for prior infarction, then further evaluation with a cardiac MRI is warranted, as it provides essential information on the characteristics of fatty myocardial lesions [3,4].

Regarding computed tomography, cardiac lipomas are well-defined hypodense lesions that do not enhance with contrast [7]. Similarly, cardiac magnetic resonance imaging has significant utility in characterizing cardiac tumors based on their signal characteristics [8]. Because of their fatty composition, cardiac lipomas usually show homogeneous increased signal intensity on T1-weighted imaging and have a complete signal loss on fat suppression sequences although thin septations are sometimes visualized [8,9]. Furthermore, the black boundary sign, due to the chemical shift effect on gradient recalled echo sequences, including b-SSFP, can help identify and correctly diagnose small lipomas [3,10]. The lack of contrast enhancement along with its described radiologic features above helps distinguish lipomas from other cardiac pathologies [9]. Although lipomatous hypertrophy of the interatrial septum usually involves the interatrial septum, there have been case reports of it involving the interventricular septum [11]. This patient’s fatty lesion was localized rather than more infiltrative, which is usually the case in lipomatous hypertrophy [11]. In the case of this patient, the cardiac MRI protocol was oriented toward infarct and not mass evaluation based on the patient’s clinical history and CT findings prior to MRI. Thus, dedicated T1-weighted images were not obtained. However, we demonstrate the utility of b-SSFP images with the “black boundary” effect along with the bright signal on the LGE sequences to confirm the fatty nature of these lesions. Additionally, the LGE sequences demonstrating preservation of the dark subendocardium along with the lack of any other bright LGE-related signal outside the fatty lesion helped to support the diagnosis. Lastly, the lack of regional wall motion provided more imaging findings for intramyocardial lipoma.

Transthoracic echocardiogram is often highlighted in reports of cardiac lipomas for its utility in the detection of suspected cardiac tumors, including their size, location, and apparent relationship to adjacent structures [1]. Classically, intracavitary lipomas are homogeneous hyperechoic lesions while pericardial lesions appear hypoechoic [3,9]. However, limitations exist with TTE when attempting to distinguish between different primary tumors of the heart thus highlighting the importance of further imaging with CT or usually MRI [2,3].

Through 2020, approximately 400 cases of cardiac lipoma have been described in the medical literature without guidelines on the best treatment plan to pursue, especially for asymptomatic patients [1,3,7]. It is widely regarded that symptomatic lipomas should be referred to cardiac surgery for resection [1-3]. With low morbidity and mortality associated with tumor resection, it is reasonable to offer such treatment modality to all patients with disease due to sequelae associated with lipoma growth, although the literature has more subepicardial and subendocardial lipomas in the surgical reviews with intramyocardial lesions likely being more challenging surgically [4,7,12]. In a review of cardiac lipomas by Shu et al., 68.3% of asymptomatic patients underwent surgical resection versus observation with follow-up [3]. However, for patients who have small asymptomatic lesions, a conservative approach can be adopted [4]. This case report aims to enhance understanding of diagnostic cardiac MRI criteria for intramyocardial lipomas along with management insights into these subtypes of cardiac lipomas.

## Conclusions

Cardiac lipomas are benign primary tumors of the heart that are commonly diagnosed incidentally. While most diagnostic evaluations of cardiac lipomas begin with TTE, these lesions are increasingly detected incidentally on CT with cardiac MRI being the imaging modality of choice to best characterize these tumors and understand their relationship with adjacent structures within the heart. For intramyocardial lipomas, we highlight the utility of b-SSFP images in confirming the fatty nature of these lesions while using the “black boundary” effect. Although fat and scar are both bright on LGE sequences, we review key findings of normal subendocardial dark signal and lack of any bright signal outside the fatty lesion that make the diagnosis of intramyocardial lipoma over post-infarct metaplasia. Normal wall motion was the last imaging finding that favored lipoma over infarction. Lastly, this case supports the conservative management of intramyocardial lipomas that are located distally since this patient was free of any adverse arrhythmias for at least six years.
